# Quality of life aspects of intermittent catheterization in neurogenic and non-neurogenic patients: a systematic review on heterogeneity in the measurements used

**DOI:** 10.1177/17562872241303447

**Published:** 2024-12-23

**Authors:** Tess van Doorn, Rosa L. Coolen, Jan Groen, Jeroen R. Scheepe, Bertil F. M. Blok

**Affiliations:** Department of Urology, Erasmus Medical Center, Dr Molewaterplein 40, 3015 GD Rotterdam, The Netherlands; Department of Urology, Erasmus Medical Center, Rotterdam, The Netherlands; Department of Urology, Erasmus Medical Center, Rotterdam, The Netherlands; Department of Urology, Erasmus Medical Center, Rotterdam, The Netherlands; Department of Urology, Erasmus Medical Center, Rotterdam, The Netherlands

**Keywords:** intermittent catheterization, neurogenic bladder dysfunction, non-neurogenic bladder dysfunction, patient-reported outcome measure, quality of life

## Abstract

**Background:**

Clean intermittent catheterization (CIC) is the golden standard in patients with lower urinary tract dysfunction, leading to bladder emptying problems, due to neurogenic or non-neurogenic causes. CIC affects patient Quality of Life (QoL) both positively and negatively.

**Objectives:**

The aim of this systematic review is to determine which measurements are used to report on the QoL of patients who are on CIC in the currently available literature, to determine the overall QoL of patients who are on CIC and lastly, to determine whether QoL in patients who are on CIC is dependent on the underlying cause (neurogenic vs non-neurogenic).

**Design:**

This systematic review was conducted following the guidelines of the Preferred Reporting Items for Systematic Reviews and Meta-Analyses statement.

**Data sources and methods:**

The Embase, Medline, Web of Science Core Collection, CINAHL, Google Scholar, and the Cochrane CENTRAL register of trials databases were systematically searched for relevant publications until March 2023.

**Results:**

A total of 4430 abstracts were screened and 43 studies were included. Studies were published between 1993 and 2022 and consisted of only neurogenic patients in 22 studies, the others included a mixed population. The included patient populations and the used measurements/tools were heterogeneous. There were 21 measurements/tools used to measure QoL, of which 3 were not validated. One questionnaire was developed to measure QoL in patients on CIC (intermittent self-catheterization questionnaire). Other measurements were suitable for general health-related QoL, to evaluate neurogenic bladder symptoms or incontinence oriented.

**Conclusion:**

The 43 included studies showed a great variety of used tools to measure QoL in patients on CIC due to neurogenic and non-neurogenic causes. Because of lacking uniformity of the measured aspects of QoL, the different included studies could not be compared and subgroup analysis was not performed. Recommendations for future research and practice are provided.

**Trial registration:**

This systematic review was registered and published beforehand at Prospero (CRD42020181777; https://www.crd.york.ac.uk/prospero).

## Introduction

There are millions of people worldwide who suffer from lower urinary tract dysfunction (LUTD) due to various underlying conditions. Often, the cause is either an unknown (idiopathic) condition or a more evident neurological disease such as spinal cord injury (SCI) or multiple sclerosis (MS). LUTD can lead to problems with emptying the bladder, resulting in urinary retention or clinically significant post-void residual (PVR).^1,2^ If left untreated, this urinary retention or PVR could lead to infections, stone formation, and ultimately kidney damage and renal failure. One of the preferred treatments for bladder emptying is clean intermittent self-catheterization (clean intermittent catheterization (CIC)), or third-party catheterization if a patient is not able to perform CIC him/herself. Patients or their caregivers are recommended to administer CIC 4–6 times per 24 h, allowing the catheterized volume to stay below 400–500 mL to prevent the bladder from overstretching.^3,4^ Performing CIC can have a great impact on a patient’s quality of life (QoL) and has an impact on the activities of daily living of patients or their caregivers, social relationships, and cultural contexts.^5^

There are various ways to measure and report QoL, which makes it difficult to interpret or compare results between different studies in patients on CIC. Standardized use of validated tools can help to improve outcome measures and improve the generalizability of those outcomes. This enables researchers and clinicians to uniformly interpret the measured QoL of patients on CIC. The first step is to make an overview of the used tools and overall measured QoL.

Therefore, the aim of this systematic review is to evaluate the currently available literature and to determine which outcome measures are used to report on QoL in patients who perform CIC and to establish whether they are validated for this use. In addition, we will assess if the overall QoL of patients who are on CIC can be determined. Our last aim is to determine whether QoL is dependent on the underlying cause (neurogenic vs non-neurogenic) for CIC.

## Materials and methods

### Study registration

This systematic review was performed according to the Cochrane Handbook for Systematic Reviews and intervention and the results are reported according to the Preferred Reporting Items for Systematic Reviews and Meta-analyses (PRISMA) statement. Before the literature search was started, the study protocol was registered and published on Prospero (CRD42020181777; https://www.crd.york.ac.uk/prospero).

### Literature search

The Embase, Medline, Web of Science Core Collection, CINAHL, Google Scholar, and the Cochrane CENTRAL register of trials databases were systematically searched for relevant publications until March 2023. No date restrictions were applied and duplicates were removed. The complete search string can be found in the Attachment 1 (Supplemental Material). The selection process is described using the PRISMA flow diagram.^6^

### Eligibility criteria

Our aim was to include all publications of original studies concerning patients on CIC that use a QoL measurement. Studies that included patients ⩾12 years of age on chronic CIC (>3 months) due to a neurogenic or a non-neurogenic cause were found eligible. Studies on CIC for dilatation of the urethra, or studies that included patients with any form of urinary diversion were excluded. Other exclusion criteria were: the use of non-specified measurements for QoL, mixed populations included and the eligible population accounted for <90%, or if data on them were not reported separately, studies with less than 10 patients, case reports, review articles, non-English language studies, conference abstracts, or if the full-text article was not available.

### Selection of studies

Titles and abstracts were screened in Endnote independently by two researchers (T.D. and J.G./R.L.C.). Studies that were found eligible for full-text retrieval were compared between the two reviewers, and discrepancies were discussed individually. Full-text screening of the potentially eligible publications was performed by the same reviewers using a standardized screening form. Any disagreements were resolved by a third reviewer (B.F.M.B.).

### Data extraction

The predefined data were extracted from the included full-text publications by two reviewers (T.D. and J.G.) independently and documented in a standardized form. Again, any disagreements between the two reviewers were resolved by the third reviewer (B.F.M.B.). Data extracted from the articles included: study characteristics, number of included patients, sex, age, underlying cause for CIC, duration of CIC, mean age at the time of the start of CIC, frequency of CIC per 24 h, self or third-party catheterization, frequency of urinary tract infections (UTIs), comorbidities reported, whether or not patients with diversions were included, type of catheter, single-use/reusable catheter, the used tool/measurement for measuring QoL, whether or not the tool was validated, measured QoL, differences in QoL in case of follow-up, and whether or not any predictors of QoL were named.

### Outcome measurements

The primary outcome consists of the used measurements for assessing QoL and establishing the validation for the specific patient population. Secondary outcome measurements are measured QoL, measured QoL related to type of catheter used, and QoL of neurogenic versus non-neurogenic patients.

### Subgroup analyses

The following subgroups of interest were predefined: male versus female, pediatric versus adolescent patients, neurogenic versus non-neurogenic causes for catheterization, and urethral versus non-urethral (continent catheterizable tubes/stoma’s) catheterization. Analyses of subgroups were performed if the data available was sufficient to do so.

### Risk of bias assessment

The risk of bias analysis of each included study was performed by using the Cochrane Risk of Bias Assessment tool.^7^ The following items that might have led to bias were assessed: random sequence generation (selection bias), allocation concealment (selection bias), blinding of participants and personnel (performance bias), blinding of outcome assessment (detection bias), incomplete outcome data (attrition bias), selective reporting (reporting bias), other bias, a priori protocol, and consecutive study participants. A list of predefined possible confounders was developed and agreed on by the authors of this systematic review: age, gender, underlying cause/disease for CIC, comorbidities, mobility/physical disability, accessibility/reimbursement of catheters, and duration of CIC. The mentioned possible points of bias or confounders were scored as “low,” “unclear,” or “high.” The risk of bias was scored high if that specific type of bias could have influenced the reported results. The confounders possibly leading to bias were classified as high if no adjustments were made in the analysis of the results or in the protocol, or if there was an imbalance between groups for that specific aspect. The analysis was performed by two researchers (T.D. an R.L.C.). Any disagreements were resolved by a third reviewer (B.F.M.B.). The Review Manager version 5.3 of Cochrane Collaboration was used to make the figure describing the risk of bias.

## Results

### Search results

**Figure 1.**
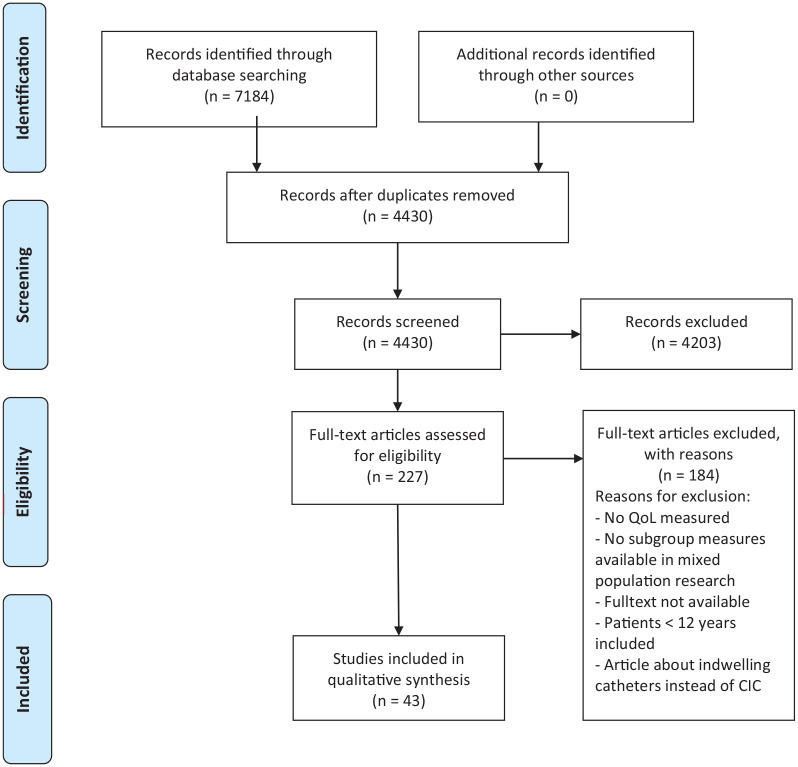
PRISMA 2009 flow diagram. Source: Moher et al.^6^ For more information, visit www.prisma-statement.org PRISMA, Preferred Reporting Items for Systematic Reviews and Meta-Analyses.

The PRISMA flow diagram is shown in Figure 1, which shows the results of the literature search and study selection. The initial search resulted in 7184 articles and after duplicates were removed, 4430 articles were left to screen. Title and abstract selection resulted in 227 articles for full-text evaluation. A total of 43 studies^5,8–49^ were included in this systematic review. Reasons for excluding studies after the full-text articles were screened, are named in the flow chart.

### Characteristics of included studies and study population

#### Study characteristics

In Table 1, the characteristics of the 43 included studies can be found. The studies included were published between 1993 and 2022 and have QoL in patients on CIC as (one of their) outcome measures. Most studies were multicenter studies (*n* = 24) or mono-centric (*n* = 12) and seven were cohort studies where participants were derived from several databases. One randomized crossover study met the inclusion criteria of this systematic review. The follow-up, shown in Table 2, was reported in 17 studies and was not applicable in 26 studies due to the type of study procedure. The duration of follow-up varied between 1 month and 7 years. All studies used the same questionnaire/tool to measure QoL for follow-up and all but two reported on the change over time; these results can be found in Table 3.

**Table 1.** Characteristics of included studies.

Article	Country	Year of publication	Type of study/design	Period of inclusion	Number of patients	Underlying cause for CIC	Duration of CIC	Sex (M/F)	Mean age (years)
Abidfaheem et al.	India	2020	Descriptive comparative study Prospective, mono center	June to December 2019	CIC *n* = 16	SCI	NR	16 Male	NR for CIC Overall 35.5 ± 10.02
Adriaansen et al.	The Netherlands	2017	Cross-sectional multicenter study	November 2011 to February 2014	*n* = 282 CIC *n* = 120	SCI	NR	90 Male 30 Female	NR for CIC Overall 48.3 ± 8.9
Akkoç et al.	Turkey	2013	Cross-sectional multicenter study	NR	Self CIC *n* = 79 Third-party CIC *n* = 65	SCI	NR	Self CIC: 68 Male 11 Female Third-party: 44 Male 21 Female	Self CIC: 37.9 ± 13.1 Third-party CIC: 35.9 ± 14.6
Bakke et al.	Norway	1993	Longitudinal multicenter study	February to August 1988	*n* = 407	106 SCI above conus 141 Lesion conus or peripheral nerves 136 Bladder dysfunction unknown etiology 19 Intravesical obstruction normal detrusor 5 Suprapontine lesions	Mean 18 months Range (0–126)	206 Male 201 Female	51.7 (Range 16–84)
Bakke and Malt	Norway	1993	Longitudinal multicenter study	NR	*n* = 302	81 SCI above conus 112 Lesion conus or peripheral nerves 100 Bladder dysfunction unknown etiology 6 Intravesical obstruction normal detrusor 3 Suprapontine lesions	Mean 20 months (range 0–127)	149 Male 153 Female	Men 54.3 Women 48.4
Bakke and Malt	Norway	1998	Prospective multicenter study	January to June 1988	*n* = 170	54 SCI above conus 61 Lesion conus or peripheral nerves 52 Bladder dysfunction unknown etiology 2 Intravesical obstruction normal detrusor 1 Cerebellar dysfunction	Mean 105 months (84–172)	84 Male 86 Female	56.9 (Range 23–89)
Banakhar et al.	Canada	2021	Prospective single-center study	NR	*n* = 16	SCI	NR	13 Male 3 Female	47.61 ± 13.9
Bolinger and Engberg	United States	2013	Cross-sectional multicenter survey	NR	*n* = 44	35 Neurogenic bladder 9 Non-neurogenic causes	Median 60 months	18 Male 26 Female	56.6 ± 16.0
Böthig et al.	Germany	2012	Mono-centric, retrospective study	NR	*n* = 56 CIC *n* = 12	Tetraplegic respiratory device-dependent SCI patients	NR	8 Male 4 Female	Mean 31.8 ± 7.89 Median 30.0 (95% CI 21.42–42.08)
Cameron et al.	United States	2011	Inception cohort study	1973/November 1995 to 2006	*n* = 7684 CIC *n* = 2914	SCI	NR	2304 Male 610 Female	NR
Castel-Lacanal et al.	France	2013	Prospective single-center study	September 2007 to July 2009	*n* = 23	MS	At start and after 9.3 ± 3 months	8 Male 15 Female	49.3 ± 10.3
Chartier-Kastler et al.	France Sweden Germany Norway Denmark	2013	Prospective, nonblinded, randomized, multicenter two-way crossover study	NR	*n* = 118	53 SCI 4 Spinal tumor 3 Spina bifida 19 MS 39 Other	Mean 10.1 ± 8.1 years Median 8.2 years	103 Male 15 Female	53.8 (22.6–87.5)
Chiappe et al.	France	2016	Cross-sectional multicenter study	November 2012 to September 2013	*n* = 171	64 SCI (42%) 25 Other neuropathies (15%) 42 Obstructive uropathy (26%) 21 MS (13%) 7 Spina bifida (4.3%)	10.5 Years 95% CI 8.93–12.1	Male 58.8% (95% CI 51.2%–66.4%)	53.8 ± 2.48
Cobussen-Boekhorst et al.	The Netherlands	2016	Prospective multicenter study	March 2012 to March 2013	*n* = 129	23 Neurogenic bladder dysfunction (18%) 70 Non-neurogenic PVR (54%) 19 Retention (15%) 6 Diabetes mellitus (5%) 11 Other (9%)	Starting, ⩾3 months	72 Male 57 Female	62 ± 13.4 (22–86)
Crescenze et al.	United States Canada	2019	Cross-sectional multicenter study with prospective enrollment	January 2016 to June 2017	*n* = 753	SCI	Median 9.5 years (range 0–44)	505 Male 248 Female	43.2 (18–86)
Elliott et al.	United States Canada	2019	Multicenter, prospective observational study	January 2016 to June 2017	*n* = 1326 CIC *n* = 879	SCI	>1 Year	352 Male 527 Female	42.7 (32.9–52.8)
Fernandez-Lasquetty Blanc et al.	Spain	2021	Prospective, multicenter observational study	October 15, 2020 to December 15, 2020	*n* = 99	16 Postoperative bladder dysfunction 27 Impaired contractile function (no neurological disorder) 32 Neurological damage 7 Neobladder 2 Infravesical obstruction (benign prostatic hyperplasia, prolapse) 10 Neurodegenerative disease (sclerosis) 2 Bladder sphincter dyssynergia 2	Starting	53 Male 46 Female	35.2 ± 20.5
Fremion et al.	United States	2021	Retrospective cohort study	NR	*n* = 88	78 Myelomeningocele 10 Non-myelomeningocele	NR	43 Male 45 Female	14–20
Fumincelli et al.	Brazil Portugal	2017	Multicenter, quantitative, cross-sectional, observational-analytic and correlational study	January 2014 to February 2015	*n* = 222 Brazil *n* = 170 Portugal *n* = 52	Brazilian patients 95 Spinal cord traumas (55.6%) 41 Acquired disease (24.0%) 27 Congenital disease (15.8%) 7 Iatrogenic disease (4.1%) Portuguese patients 41 Spinal cord trauma (78.8%) 6 Acquired disease (11.6%) 3 Congenital disease and (5.8%) 2 Iatrogenic disease (3.8%)	Starting period Brazil 1980–1989 *n* = 5 1990–2000 *n* = 23 2001–2011 *n* = 99 2012–2015 *n* = 43 Portugal 1980–1989 *n* = 1 1990–2000 *n* = 7 2001–2011 *n* = 26 2012–2015 *n* = 18	Brazil 121 Male 49 Female Portugal 38 Male 14 Female	Brazil Mean 39 Median 37 Range 18–95 Portugal Mean 45 Median 44 Range 24–83
Girotti et al.	Brazil	2011	Mono-centric, prospective follow-up study	July 2006 to May 2008	*n* = 60	30 Neurogenic cause 30 Non-neurogenic cause	Starting	39 Male 21 Female	50.4 (range 15–88)
James et al.	United States	2014	Cross-sectional study of a national registry of patients	Fall 2010	*n* = 9397 CIC *n* = 727	MS	NR	NR for CIC Overall 2126 Male 7271 Female	NR for CIC Overall 55.4 (±10.5) Range 20–93
Kessler et al.	Switzerland	2009	Mono-centric cross-sectional study	NR	*n* = 92	51 Neurogenic (55%) 13 SCI 17 MS 9 MMC 12 Peripheral bladder denervation due to radical pelvic surgery 41 Non-neurogenic (45%) 19 Anatomical/mechanical bladder outlet obstruction 22 Underactive detrusor	5 ± 6.3 Years	31 Male 61 Female	NR
Ko et al.	Korea	2022	Multicenter, open-label, observational study	NR	*n* = 360	7 Congenital spinal lesion 103 SCI 250 Other conditions	Mean 6.96 (SD 8.16) years Range: 1–42	145 Male 2015 Female	62.0 ± 13.2 Years
Leroux et al.	France	2021	Prospective, mono center study	July 2019 to October 2019	*n* = 25	19 Central neurological disease (76%) 2 Peripheral neurological disease (8%) 4 Non-neurological disease (16%)	Median 7 (3–11.5) years	11 Male 14 Female	Median: 53 (47–57) years
Liu et al.	United Kingdom	2010	Mono-centric cross-sectional study	NR	*n* = 142 Self CIC *n* = 35 Third-party CIC *n* = 11	SCI	NR	NR for CIC Overall 105 Male 37 Female	NR for CIC Overall 45.2 ± 14.6
Liu et al.	United States	2015	Mono-centric cross-sectional study	NR	Overall *n* = 66 CIC *n* = 42	Spina bifida	NR	NR for CIC Overall 22 Male 44 Female	NR for CIC Overall 32.3 ± 7.2
Luo et al.	China	2012	Cross-sectional face-to-face survey	2010	Overall *n* = 180 CIC *n* = 11	SCI	NR	NR for CIC Overall 98 Male 82 Female	NR
Markiewicz et al.	United States	2020	Cross-sectional survey	2017–2018	*n* = 384	308 SCI 20 MS 25 Spina bifida 26 Other	*n* = 10, <6 months *n* = 20, 6–12 months *n* = 31, 1–2 years *n* = 323, >2 years	247 Male 137 Female	NR (51% between 51 and 70 years)
Markiewicz et al.	United States The Netherlands	2021	Cross-sectional survey	September 2019 to March 2020	*n* = 784	211 Neurogenic/neurological disorder 573 Non-neurogenic reason	72% >1 Year	314 Male 426 Female	83% >50 Years Only age subcategories are reported
McClurg et al.	United Kingdom	2019	Mixed prospective longitudinal cohort study and retrospective survey of a national register	May 2013 to April 2014	Overall *n* = 204 CIC *n* = 43	MS	Starting	12 Male 31 Female	NR
Myers et al.	United States Canada	2019	Prospective observational study of an international registry	NR	Overall *n* = 1479 CIC *n* = 754	SCI	NR	NR for CIC Overall 894 Male 585 Female	NR for CIC Overall: 44.9 (IQR 34.4–54.1)
Newman et al.	United States Australia	2020	Cross-sectional cohort study with subsequent prospective multicenter 4-week clinical trial	June 2014 to May 2017	*n* = 39	26 NLUTD (67%) 13 LUTD (33%)	Mean 10 ± 9 years, median 7 years	27 Male 12 Female	Mean 55 ± 13 Median 56 Range 28–82
Oh et al.	South Korea	2005	Prospective, multicenter trial	March 2002 to February 2003	*n* = 132	SCI	24.2 ± 3.1 Months	81 Male 51 Female	41.8 ± 1.4
Pinder et al.	United Kingdom France Germany	2012	Cross-sectional multicenter validation study	NR	*n* = 306	Neurogenic bladder (SCI/MS)	118.5 ± 147.8 Months, range 6–420	202 Male 104 Female	Mean 46.1 ± 12.4 Range 19–78
Roberson et al.	United States	2021	Cross-sectional multicenter clinical study	September 2016 to May 2019	*n* = 200	109 SCI 46 Retention/residu 10 Urethral obstruction/bladder neck contracture 15 Spina bifida 5 MS 5 Postoperative retention 3 Augmentation/catheterizable channel 2 Transverse myelitis 5 Other	*n* = 22, 6–12 months *n* = 45, 13 months to 3 years *n* = 35, 3.1–5 years *n* = 98, >5 years	140 Male 60 Female	Median 51 Range 19–90
Sekido et al.	Japan	2021	Prospective Cross-sectional single-center study	September 1997 to August 2011	*n* = 50	Urinary retention after radical hysterectomy	<24 Months *n* = 11 ⩾24 Months *n* = 39	Only female	Mean 49.3 ± 11.9 years
Svihra et al.	Slovakia	2018	Prospective multicenter study	January to June 2014	*n* = 229	SCI	⩾6 Months	172 Male 57 Female	Mean 46.6 ± 31.5 Range 18–81
Wilde et al.	United States	2016	Prospective mono-centric intervention study	NR	*n* = 29	SCI	NR	15 Male 14 Female	Mean 43.52 ± 13.10 Median 47
Yasami et al.	Iran	2017	Mono-centric cross-sectional study	September 2012 to August 2014	Overall *n* = 171 Self CIC *n* = 46 Third-party CIC *n* = 41	SCI	NR	NR for CIC Overall: 135 Male 36 Female	NR for CIC Overall: Mean: 47.57 ± 14.87 Men: 47.04 ± 14.98, Women : 49.56 ± 14.48
Yeşil et al.	Turkey	2020	Cross-sectional, multicenter validation study	NR	*n* = 60	SCI	Total group: 36.38 ± 35.1 months Men: 34.35 ± 33.62 months Women: 40.45 ± 38.58 months	40 Male 20 Female	Total group: 37.07 ± 12.6 Men: 38.08 ± 13.51 Women: 35.05 ± 10.61
Yilmaz et al.	Turkey	2014	Cross-sectional multicenter study	NR	*n* = 269	SCI	Mean: 48 months Men: 45.95 ± 44.25 months (range 3–340) Women: 54.95 ± 50.20 months (range 3–192)	199 Male 70 Female	Total group: 41.11 ± 13.95 Men: 40.73 ± 13.85 Women: 42.20 ± 14.25
Yoshida et al.	Japan	2017	Cross-sectional two-center validation study	October 2013 to April 2015	*n* = 70	61 Neurogenic bladder 9 BPH	7.6 ± 9.1 Years	30 Male 40 Female	62.8 ± 17.7
Zanollo et al.	Norway France Italy	2015	Cross-sectional multicenter study, with historical control cohort	NR	Study group *n* = 84 Control group *n* = 316	SCI	Control group, all but one ⩾3 months	Control group male 68%/female 32% Study group male 72%/female 28%	NR

BPH, Benign prostate hyperplasia; CIC, clean intermittent catheterization; LUTD, lower urinary tract dysfunction; MMC, Meningomyocele (spina bifida); MS, multiple sclerosis; NLUTD, Neurogenic Lower Urinary Tract Dysfunction; NR, not reported; PVR, post-void residual; SCI, spinal cord injury.

**Table 2.** Characteristics of included studies.

Article	Mean age at start of CIC (years)	Frequency CIC/24 h	Self or third-party catheterization	UTI	Comorbidities	Diversion	Type of catheter	Single-use/reusable	Follow-up
Abidfaheem et al.	NR	NR	NR	5 Patients, 31.3%	NR for CIC	NR	NR	NR	NA
Adriaansen et al.	NR	NR	113 Self CIC 7 Third-party CIC	NR	NR	Excluded	NR	NR	NA
Akkoç et al.	NR	NR	79 Self CIC 65 Third-party CIC	NR	NR	NR	NR	NR	NA
Bakke et al.	NR	NR	268 Self CIC 31 Third-party CIC	0.6 (Range 0–6) over 3 months, combined frequency and severity score	NR	NR	95.8% Low friction (LoFric; Wellspec)	Disposable sterile PVC Single-use	Mean 13 months
Bakke and Malt	NR	NR	NR	0.59 (0–6)	32.8% (*n* = 99) Comoborbidity without major disability like MS or DM 30.8% (*n* = 93) Severe comorbidity such as paraplegia, cancer, or disabling MS	NR	NR	Disposable sterile PVC single-use	13.2 Months (range 9–17 months)
Bakke and Malt	NR	NR	NR	NR	NR	NR	91% Lofric, one patient plain PVC catheter	Disposable catheters 1 Patient reusable glass catheter	7 Years
Banakhar et al.	NR	NR	NR	NR	NR	Analyzed separately	NR	NR	NA
Bolinger and Engberg	NR	NR	Only self CIC	NR	NR	Excluded	NR	57% Reusable, median 14 times	NA
Böthig et al.	NR	NR	Only third party	NR	NR	Analyzed separately	NR	NR	NA
Cameron et al.	NR	NR	NR	NR	NR	Excluded	NR	NR	NA
Castel-Lacanal et al.	49.3 ± 10.3	NR	NR	NR	NR	NR	NR	NR	9.3 ± 3 Months
Chartier-Kastler et al.	NR	Mean 5.6/24 h	NR	During follow-up two UTIs in two individuals reported	NR	NR	Compact hydrophilic-coated catheter (Sppedicath compact)	Single-use	84 ± 6 Days (crossover after 42 ± 3 days)
Chiappe et al.	NR	4.84/24 h 95% CI 4.53–5.15	NR	UTI over last year according to GP (*n* = 171) *n* = 100 (63.3%) According to patient (*n* = 119) *n* = 78 (65.5%)	None *n* = 50 (27%) Arterial hypertension *n* = 38 (20.5%) Anxiety *n* = 33 (17.8%) Arthrosis *n* = 16 (8.65%) Diabetes *n* = 10 (5.41%) Cardiac insufficiency *n* = 10 (5.41%) Cancer *n* = 9 (4.86%) COPD *n* = 5 (2.70%) Stroke *n* = 4 (2.16%) Gastric ulcer *n* = 2 (1.08%) Osteoporosis *n* = 2 (1.08%) Rheumatic disease *n* = 1 (0.54%)	NR	*n* = 111 Coloplast *n* = 70 (63.1%) Speedicath *n* = 45 Easycath *n* = 11 Wellspect Lofric *n* = 18 (16.2%) B.Braun Actreen *n* = 10 (9.01%) Advance Hollister *n* = 7 (6.31%) Other *n* = 6 (5.41%)	Single-use	NA
Cobussen-Boekhorst et al.	62 ± 13.4 (22–86)	3.3 (0.3–10) Times/24 h	120 Self CIC 9 Third-party CIC	28 (47%), No infection/3 months 19 (32%) ⩾1 Antibiotics/year 13 (22%) Daily maintenance dose antibiotics	Obesity *n* = 8 (6%) Prolapse *n* = 2 (2%) Spasm *n* = 3 (2%) BPH *n* = 2 (2%) Visual impairment *n* = 5 (4%) Age *n* = 2 (2%) Cognitive impairment *n* = 3 (2%) Chronic bronchitis/COPD *n* = 3 (2%) Other *n* = 6 (5%) None *n* = 95 (71%)	NR	NR (based on patients preference)	Single-use	1 Year
Crescenze et al.	NR	NR	82 (10.9%) Third-party CIC	>4 UTIs/year in 209 (27.8%) patients	NR	Analyzed separately	NR	NR	NA
Elliott et al.	NR	NR	111 (13%) Third-party CIC	NR	Charlson comorbidity score 0: *n* = 791 (88.2%) 1: *n* = 60 (6.8%) ⩾2: *n* = 28 (3.2%)	Analyzed separately	NR	NR	NA
Fernandez-Lasquetty Blanc et al.	35.2 ± 20.5	NR	67 (67.7%) Self CIC 14 (15.7%) Third-party CIC	NR	Obesity *n* = 17 (17.2%) Prolapse *n* = 1 (1.1%) Benign prostate hyperplasia *n* = 10 (10.1%) Bladder spasm *n* = 1 (1.1%) History of depression *n* = 12 (12.1%) History of anxiety *n* = 11 (11.1%)	NR	Hydrophilic: *n* = 13 (13.1%) Pre-lubricated: *n* = 66 (66.6%)	NR	1 Month
Fremion et al.	NR	NR	NR	NR	NR	Urethral CIC *n* = 53 (60.2) Channel CIC *n* = 24 (27.3) But not analyzed separately	NR	NR	Median 6.5 months (range 0.0–3.1)
Fumincelli et al.	NR	Brazil (*n* = 170) 2 Times/24 h *n* = 8 (4.7%) 3 Times/24 h *n* = 17 (10%) 4 Times/24 h *n* = 78 (45.9%) 5 Times/24 h *n* = 33 (19.4%) 6 Times/24 h *n* = 25 (14.7%) 7 Times/24 h y *n* = 4 (2.4%) 8 Times/24 h *n* = 5 (2.9%) Portugal (*n* = 52) 2 Times/24 h *n* = 2 (3.8%) 3 Times/24 h *n* = 1 (1.9%) 4 Times/24 h *n* = 15 (28.8%) 5 Times/24 h *n* = 11 (21.2%) 6 Times/24 h y *n* = 16 (30.8%) 7 Times/24 h *n* = 7 (13.5%)	Self CIC Brazil *n* = 107 (62.9%) Portugal *n* = 48 (92.3%)	NR	NR	NR	Brazil Simple *n* = 157 (92.4%) Glass *n* = 12 (7.1%) Lubricated *n* = 1 (0.6%) Portugal Lubricated *n* = 50 (96.2%) Silicon *n* = 2 (3.8%)	NR	NA
Girotti et al.	50.4	NR	NR	18/60 Patients in 12 months	NR	NR	NR	NR	1 Year
James et al.	NR	NR	NR	NR	NR	NR	NR	NR	NA
Kessler et al.	50 ± 18	3 ± 2 Times/24 h	NR	NR		Excluded	NR	NR	NA
Ko et al.	NR	Nr of catheters used/24 h ⩽1: *n* = 6 (5.4%) 2: *n* = 16 (14.4%) 3: *n* = 13 (11.7%) 4: *n* = 28 (25.2%) 5: *n* = 16 (14.4%) 6: *n* = 13 (11.7%) 7: *n* = 9 (8.1%) 8: *n* = 5 (4.5%) ⩾10: *n* = 3 (2.7%)	NR	9.72%	NR	NR	At inclusion Silicone *n* = 48 (46.6%) Latex *n* = 40 (36%) Polyvinyl chloride *n* = 2 (1.8%) Polyvinyl pyrrolidone *n* = 1 (0.9%) Unknown *n* = 20 (18%) During study: SpeediCath (hydrophilic)	Single use	24 Weeks
Leroux et al.	NR	Numbers of CIC/24 h <4: *n* = 2 (8%) 4–6: *n* = 8 (32%) >6: *n* = 15 (60%)	Only self CIC	NR	NR	NR	NR	NR	NA
Liu et al.	NR	NR	35 Self CIC 11 Third-party CIC	NR	NR	NR	NR	NR	NA
Liu et al.	NR	NR	NR	NR	NR	Analyzed separately	NR	NR	NA
Luo et al.	NR	NR	NR	9 of 11 Patients, 81.82%	NR	NR	NR	NR	NA
Markiewicz et al.	NR	NR	315 Self CIC 69 Third-party CIC	NR	NR	NR	NR	NR	NA
Markiewicz et al.	NR	NR	NR	39.4% Reported a UTI 6 months prior to study	NR	NR	Discreet catheter	NR	NA
McClurg et al.	49.9 ± 12.5	NR	NR	NR	NR	NR	45% Speedicath 31% LoFric 16% Actreen 7% Hydrophilic	Single-use	1 Year
Myers et al.	NR	NR	NR	NR	NR	Exluded	NR	NR	NA
Newman et al.	NR	Mean 6 ± 2×/24 h, median 6 (range 4–11)	NR	6 UTIs in total during 4-week follow-up	NR	NR	During trial, hydrophilic-coated catheters At inclusion of silicone, latex/rubber, or plastic catheters	Single-use during trial, reusable at inclusion	1 Month
Oh et al.	NR	NR	88 Self CIC 44 Third-party CIC	NR	NR	NR	NR	NR	NA
Pinder et al.	NR	NR	Only self CIC	NR	NR	NR	NR	NR	2 Months
Roberson et al.	NR	Frequency of CIC/24 h 0–1: *n* = 12 (6%) 2: *n* = 6 (3%) 3: *n* = 12 (6%) 4: *n* = 36 (18%) 5: *n* = 57 (28.5%) 6: *n* = 45 (22.5%) >6: *n* = 32 (16%)	NR	Nr of UTIs treated in the 6 months prior No: *n* = 96 (48%) 1: *n* = 43 (21.5%) 2: *n* = 25 (12.5%) 3: *n* = 9 (4.5%) 4: *n* = 7 (3.5%) Do not know: *n* = 20 (10%)	NR	NR	PVC: *n* = 58 (29%) Catheter with gel lubricant: *n* = 42 (21%) Hydrophilic (water sachet): *n* = 35 (17.5%) Hydrophilic (fluid coating): *n* = 29 (14.5%) Red rubber: Other: *n* = 16 (8%) Did not know: *n* = 7 (3.5%)	Single use: *n* = 184 (92%) Reusable: *n* = 16 (8%)	NA
Sekido et al.	44.7 ± 10.6	NR	Only self CIC	NR	Diabetes mellitus *n* = 1 Hypoparathyroidism *n* = 1 Lumbar spondylosis *n* = 1	NR	NR	NR	NA
Svihra et al.	NR	Mean 4.9 ± 1.8, range 1–11	191 Self CIC 38 Third-party CIC	NR	NR	NR	NR	NR	6 Months
Wilde et al.	NR	Baseline 2–9×/24 h, after follow-up 4–6×/24 h	Only self CIC	0.65 ± 0.94 UTIs per patient in previous 3 months After 3 months 0.45 ± 0.74 UTIs	NR	NR	Plastic not coated *n* = 10 Hydrophilic *n* = 5 Silicone *n* = 3 Latex *n* = 3 Unknown *n* = 7	NR	3 Months
Yasami et al.	NR	NR	46 Self CIC 41 Third-party CIC	NR	No, exclusion criteria	NR	NR	NR	NA
Yeşil et al.	NR	NR	NR	NR	NR	NR	NR	NR	NA
Yilmaz et al.	NR	NR	69.5% Self CIC 30.5% Third-party CIC	NR	None	NR	75.7% Pre-lubricated 19.9% Hydrophilic 4.5% Other types	NR	NA
Yoshida et al.	NR	145 (54%) 6×/24 h	NR	NR	NR	NR	NR	Single-use *n* = 14 Reusable *n* = 56	1 Month (*n* = 30)
Zanollo et al.	NR	NR	Control group 85% Self CIC 15% third-party CIC Study group 85% self CIC 15% third-party CIC	NR	NR	NR	NR	NR	1 Year

CIC, clean intermittent catheterization; COPD, Chronic Obstructive Pulmonary Disease; DM, Diabetes Mellitus; MS, multiple sclerosis; UTI, urinary tract infection.

**Table 3.** Characteristics of included studies.

Article	Used tool/measurement QoL	Pro’s tool	Cons tool	Validated yes/no	Measured QoL start	Measured QoL end of follow-up	Differences in QoL	Predictors QoL
Abidfaheem et al.	Qualiveen 30	NR	NR	Yes	SF-Qualiveen Bother with limitations: 1.589 ± 0.49 Frequency of limitations 1.945 ± 0.46 Fears: 2.026 ± 0.51 Feelings: 1.497 ± 0.53 Overall QoL: 1.746 ± 0.39	NA	NA	NR
Adriaansen et al.	SF-Qualiveen	NR	NR	Yes	Self CIC 1.29 (±0.65) Third-party CIC 1.48 (±0.91)	NA	NA	NR
Akkoç et al.	King’s Health Questionnaire	NR	NR	Yes	Self CIC GHP 33.3 ± 21.5 II 55.9 ± 35.5 RL 41.3 ± 35.2 PL 40.0 ± 33.8 SL 43.8 ± 35.7 PR 45.4 ± 35.8 EM 33.6 ± 27.3 S/E 23.7 ± 25.1 SS 41.6 ± 27.3 Third-party GHP 41.6 ± 28.2 II 61.3 ± 34.9 RL 53.4 ± 36.6 PL 57.5 ± 35.7 SL 49.6 ± 34.6 PR 52.1 ± 34.9 EM 45.9 ± 33.7 S/E 26.6 ± 25.7 SS 48.8 ± 28.1	NA	NA	NR
Bakke et al.	GHQ-28	NR	NR	Yes	Mean distress 25.5 at start New patient No aversion (*n* = 36) 24.0 Aversion (*n* = 13) 30.1 CIC has had advantages (*n* = 41) 23.4 CIC has had no advantages (*n* = 3) 39.0	Mean distress 24.5 using CIC previously After 1 year Patient using CIC previously No aversion (*n* = 161) 21.1 Aversion (*n* = 77) 31.0 CIC has had advantages (*n* = 182) 23.4 CIC has had no advantages (*n* = 30) 28.3	NR	Opinion on performing CIC Clinical UTI Feelings of advantages of CIC Aversion to CIC
Bakke and Malt	GHQ-28	NR	NR	Yes	Men 5.3 (4.8–5.9) Women 7.3 (6.7–7.9)	NR	NR	NR
Bakke and Malt	GHQ-28	NR	NR	Yes	Values given dependent on state of UTI at start and end of study	Values given dependent on state of UTI at start and end of study	Significant changes, dependent on state of UTI	NR
Banakhar et al.	SF-36	NR	NR	Yes	SF-36 PF 25.4 ± 12 RP 40.1 ± 8.1 BP 39.01 ± 1.8 GH 43.6 ± 10 VT 40.2 ± 8.9 SF 38.6 ± 9.3 RE 50 ± 5.6 MH 47.8 ± 12	NA	NA	NR
Bolinger and Engberg	SF-36	NR	NR	Yes	Mean SF-36 physical component 33.7 ± 12.2 Mean SF-36 mental component 50.8 ± 13.4	NA	NA	NR
Böthig et al.	ICIQ-SF	Easy and free to use	NR	Yes	Mean 4.27 (±2.80) 95% CI: 2.328–6.217 Median: 4.0	NA	NA	NR
Cameron et al.	SF-36 Perceived health status SWLS	NR	NR	Yes	SWLS 18.77 ± 0.44 Perceived Health Status 2.76 ± 0.06	NA	NA	NR
Castel-Lacanal et al	Qualiveen SF-36	NR	NR	Yes	*N* = 22 Qualiveen Overall QoL 1.63 ± 0.13 Bother with limitations 1.6 ± 0.16 Frequency of limitations 2.12 ± 0.16 Fears 1.36 ± 0.15 Feelings 1.46 ± 0.22 SF-36 General physical score 36.6 ± 2.1 General mental score 39.1 ± 2.6 PF 34.6 ± 2.7 RP 34.7 ± 2.4 BP 41.6 ± 2.5 GH 34.4 ± 1.3 VT 39.5 ± 1.5 SF 38.5 ± 2.7 RE 35.7 ± 2.8 MH 38.4 ± 2.6	*N* = 22 Qualiveen Overall QoL 1.31 ± 0.15 Bother with limitations 1.12 ± 0.19 Frequency of limitations 1.87 ± 0.21 Fears 1.09 ± 0.18 Feelings 1.09 ± 0.18 SF-36 General physical score 34.6 ± 1.9 General mental score 36.4 ± 2.8 PF 30.8 ± 3.2 RP 31.7 ± 2.3 BP 41.3 ± 2.7 GH 32.2 ± 2.3 VT 37.6 ± 2.1 SF 36.3 ± 2.4 RE 31.5 ± 3.2 MH 36.1 ± 3	*n* = 22 Qualiveen Overall QoL *p* = 0.004 Bother with limitations *p* = 0.007 Frequency of limitations *p* = 0.35 Fears *p* = 0.02 Feelings *p* = 0.02 SF-36 General physical score *p* = 0.22 General mental score *p* = 0.55 PF *p* = 0.20 RP *p* = 0.24 BP *p* = 0.84 GH *p* = 0.31 VT *p* = 0.87 SF *p* = 0.61 RE *p* = 0.24 MH *p* = 0.76	NR
Chartier-Kastler et al.	ISC-Q VAS	QoL specific to the needs of neurological patients who perform CIC	NR	Yes	Standard catheter ISC-Q 60 VAS for general satisfaction: 71 for standard catheters, 77 for Compact catheter	Compact catheter ISC-Q 76.8	ISC-Q 17.0 ± 1.8, 28% increase when using Compact catheter	NR
Chiappe et al.	Qualiveen SF-12	NR	NR	Yes	Qualiveen Overall QoL 1.38 (95% CI 1.23–1.53) Bother with limitations 1.49 (95% CI 1.31–1.67) Frequency of limitations 1.68 (95% CI 1.48–1.88) Fears 1.25 (95% CI 1.08–1.42) Feelings 1.09 (95% CI 0.89–1.29) SF-12 physical 38.6 (95% CI 36.8–40.4) SF-Mental 46.4 (95% CI 44.3–48.5)	NA	NA	NR
Cobussen-Boekhorst et al.	VAS score on QoL King’s Health score	NR	NR	Yes	VAS Score: 5.20 Kings Health GHP 42 ± 19.424 II 59.67 ± 32.925 RL 45.29 ± 34.266 PL 36.23 ± 30.523 SL 21.39 ± 23.444 PR 35.09 ± 33.877 EM 24.35 ± 23.203 S/E 35.67 ± 30.153 SS 23.75 ± 22.360	VAS Score: 6.93 Kings Health GHP 40.50 ± 18.042 II 45 ± 28.965 RL 33.51 ± 31.593 PL 29.71 ± 31.334 SL 20.06 ± 26.105 PR 31.87 ± 32.164 EM 17.51 ± 24.389 S/E 30.33 ± 28.464 SS 24.17 ± 25.692	Kings Health 95% CI GHP −2.539, 5.539 II 7.186, 22.147 RL 4.281, 19.270 PL −0.149, 13.193 SL −3.088, 5.743 PR −5.993, 12.426 EM 1.996, 11.696 S/E −1.362, 12.028 SS −5.172, 4.339	NR
Crescenze et al.	NBSS QoL SF-12	NR	NR	Yes	NBSS QoL Dissatisfied *n* = 272; 3.5 ± 0.5 NBSS QoL Neutral/Satisfied *n* = 481; 1.4 ± 0.7 Dissatisfied/NBSS total *n* = 272; 30.8 ± 12 Neutral/Satisfied/NBSS total *n* = 481; 23.1 ± 9.6 Dissatisfied/SF-12 mental *n* = 272; 47 Neutral/Satisfied/SF-12 mental *n* = 481; 52 Dissatisfied/SF-12 physical *n* = 272; 40 Neutral/Satisfied/SF-12 physical *n* = 481; 45	NA	NA	Female ⩾4 UTIs/year Bowel dysfunction
Elliott et al.	SF-12 physical + mental	NR	NR	Yes	SF-12 physical 41.8 (33.4–51.7) SF-Mental 52.2 (40.5–57.7)	NA	NA	NR
Fernandez-Lasquetty Blanc et al.	King’s Health Questionnaire			Yes	GHP 39.7 ± 21.16 II 58.9 ± 32.93 RL 37.2 ± 33.06 PL 36.5 ± 36.63 SL 29.7 ± 31.80 PR 55.7 ± 33.41 EM 28.2 ± 26.52 S/E 28.1 ± 32.61 SS 35.3 ± 26.84	GHP 36.7 ± 20.0 II 43.5 ± 33.1 RL 28.1 ± 31.4 PL 26.2 ± 31.5 SL 20.5 ± 28.6 PR 50.0 ± 30.7 EM 15.6 ± 22.7 S/E 15.4 ± 30.9 SS 29.5 ± 25.7	GHP 6.33 II 14.77 RL 8.65 PL 9.29 SL 7.03 PR 13.00 EM 11.67 S/E 14.56 SS 7.68	NR
Fremion et al.	QUALAS-T	NR	NR	Yes	QUALAS-T QFI: 73.8 ± 19.9 QBB: 63.8 ± 25.8	NR	No significant changes over time	No variables were associated with a change in QoL
Fumincelli et al.	WHOQoL-Bref	NR	NR	Yes	Brazil Physical 58.9 ± 18.8 Psychological 68.9 ± 18.4 Social 65.8 ± 21.4 Environment 61.4 ± 16.4 QoL 18.1 ± 5.5 Health 16.9 ± 6.4 Portugal Physical 60.3 ± 16.5 Psychological 68.4 ± 16.5 Social 63.4 ± 19.7 Environment 59.4 ± 13.7 QoL 16.7 ± 4.1 Health 14.2 ± 5.2	NA	NA	Improvement in urinary symptoms, independence, self-confidence, social relationships, access to work activities, and social insertion
Girotti et al.	WHOQoL-Bref	NR	NR	Yes	VAS pre-training 6.49 ± 2.18	*n* = 37 Physical 54.41 Psychological 66.30 Social relationships 63.73 Environment 54.85 Self-evaluation QoL 56.62 VAS post-training 2.38 ± 1.62	NR	NR
James et al.	Semiannual NARCOMS questionnaire 7-Point Likert scale (indicating change due to treatment)	NR	NR	No	Reported change in QoL: Positive change *n* = 387 No change *n* = 148 Negative change *n* = 192	NA	NA	NR
Kessler et al.	Questionnaire based on the validated SF-12	NR	NR	No	Changes in QoL due to CISC: Extreme improvement *n* = 32 (35%) Improvement *n* = 24 (26%) No change *n* = 25 (27%) Deterioration *n* = 7 (8%) Extreme deterioration *n* = 4 (4%)	NA	NA	Severe pain while inserting catheter
Ko et al.	ISC-Q	NR	NR	Yes	NR for total group at start	At 12-week follow-up (*n* = 264) Ease to use 67.77 ± 22.02 Convenience 59.33 ± 27.15 Discreetness 54.31 ± 25.39 Psychological 36.92 ± 21.43 Total 54.58 ± 18.16 At 24-week follow-up (*n* = 271) Ease to use 68.01 ± 22.29 Convenience 59.50 ± 26.40 Discreetness 53.98 ± 26.51 Psychological 38.12 ± 22.06 Total 54.90 ± 18.65	Mean change baseline—12 weeks Ease to use 14.33 ± 31.40 Convenience 20.19 ± 41.22 Discreetness 20.31 ± 36.12 Psychological 5.75 ± 28.14 Total 15.14 ± 25.91 Mean change baseline—24 weeks Ease to use 7.24 ± 19.96 Convenience 20.03 ± 40.55 Discreetness 19.03 ± 35.06 Psychological 9.66 ± 30.64 Total 15.58 ± 25.95	NR
Leroux et al.	SF-Qualiveen	NR	NR	Yes	Median 1.4 (0.8–2)	NA	NA	A longer CIC specific or total time was not correlated with poorer QoL (*p* = 0.43)
Liu et al.	SF-36 King’s Health Questionnaire	NR	NR	Yes	SF-36 Self CIC/Third-Party PF 16.1 ± 18.3/12.5 ± 26.7 RP 22.7 ± 33.2/12.4 ± 22.5 BP 45.1 ± 28.8/41.7 ± 14.9 GH 51.9 ± 20.6/49.8 ± 13.1 VT 53.1 ± 19.8/47.5 ± 25.6 SF 61.3 ± 30.3/62.5 ± 22.8 MH 61.2 ± 18.9/48.7 ± 23.7 RE 67.7 ± 17.7/58.1 ± 29.2 PCS 28.7 ± 6.7/24.8 ± 4.7 MCS 42.1 ± 12.1/34.3 ± 11.1 Kings Health Self CIC/Third-Party GHP 48.7 ± 25.7/52.5 ± 28.3 II 42.4 ± 26.6/50.6 ± 39.3 RL 35.8 ± 28.9/51.3 ± 21.8 PL 44.0 ± 33.4/49.5 ± 28.2 SL 44.8 ± 28.9/56.9 ± 22.5 PR 34.8 ± 35.1/48.6 ± 33.7 EM 42.1 ± 31.4/54.8 ± 28.3 S/E 35.3 ± 23.8/48.3 ± 18.3 SS 41.5 ± 15.4/45.4 ± 23.1	NA	NA	Third-party CIC Incontinence
Liu et al.	I-QoL SF-36	NR	NR	Yes	I-QoL Avoidance Behavior 52.7 ± 21.8 I-QoL Psychosocial impact 60.5 ± 26.1 I-QoL Embarassment 53.1 ± 28.3 I-QoL Sum 53.6 ± 21.3 mSF36 PF 63.5 ± 28.8 mSF36 Role Lim Phys 67.5 ± 34.7 mSF36 Role Lim Emo 73.2 ± 32.3 mSF36 Energy/Fatigue 52.8 ± 25.3 mSF36 Emotional Well-being 67.8 ± 23.7 mSF36 SF 71.4 ± 31.9 mSF36 BP 72.6 ± 28.0 mSF36 GH 55.2 ± 25.2 mSF36 Health trans 58.3 ± 22.5	NA	NA	NR
Luo et al.	WHOQoL-Bref	NR	NR	Yes	Physical health 9.89 ± 1.78 Psychological health 8.55 ± 2.03 Social relationship 9.34 ± 0.72 Environment 12.27 ± 2.10	NA	NA	NR
Markiewicz et al.	VAS	NR	NR	Yes	Male 75.04 (95% CI: 72.67–77.40) Female 76.88 (95% CI: 74.14–79.62)	NA	NA	NR
Markiewicz et al.	VAS	NR	NR	Yes	CIC interference with social life and QoL Most or all of the time: 55.1 Some to the time VAS: 67.0 Little or non of the time VAS: 74.2 CIC interference with work outside the home and QoL Most or all of the time: 57.7 Some to the time VAS: 65.6 Little or non of the time VAS: 73.9	NA	NA	NR
McClurg et al.	ICIQ-LUTS ICIQ-FLUTS EQ-5D EQ-5D VAS	NR	NR	Yes	ICIQ-LUTS 47.0 ± 11.5 ICIQ-FLUTS 18.0 ± 6.9 EQ-5D 0.64 ± 0.19 EQ-5D VAS 61 ± 14.5	ICIQ-LUTS 45.9 ± 11.2 ICIQ-FLUTS 17.8 ± 6.5 EQ-5D 0.657 ± 0.135 EQ-5D VAS 59.4 ± 8.1	NR	NR
Myers et al.	NBSS SCI-QoL difficulties Three subdomains of NBSS NBSS satisfaction	NR	NR	Yes	NBSS: Paraplegia: 26.6 (95% CI: 25.8–27.5) Tetraplegia: 23.6 (95% CI: 22.2–24.9) SCI-QoL Difficulties score: Paraplegia: 59.8 (95% CI: 59.2–60.5) Tetraplegia: 57.2 (95% CI: 56.2–58.3) NBSS Incontinence: Paraplegia: 12.2 (95% CI: 11.6–12.8) Tetraplegia: 8.6 (95% CI: 7.7–9.5) NBSS Storage and voiding: Paraplegia: 7.8 (95% CI: 7.5–8.1) Tetraplegia: 8.0 (95% CI: 7.5–8.5) NBSS Consequences: Paraplegia: 6.7 (95% CI: 6.4–6.9) Tetraplegia: 7.0 (95% CI: 6.6–7.4) NBSS Satisfaction: Paraplegia: 2.2 (95% CI: 2.1–2.3) Tetraplegia: 2.1 (95% CI: 1.9–2.3)	NA	NA	NR
Newman et al.	ISC-Q	NR	NR	Yes	Ease to use 62.66 ± 20.12 Median 59.38 Convenience 55.61 ± 30.88 Median 50.00 Discreetness 65.45 ± 27.34 Median 66.67 Psychological 48.27 ± 30.72 Median 41.67 Total 58.00 ± 22.57 Median 53.39	Ease to use 74.65 ± 19.71 Median 78.13 Convenience 60.07 ± 28.94 Median 62.50 Discreetness 76.62 ± 21.46 Median 83.33 Psychological 57.41 ± 28.97 Median 52.08 Total 67.19 ± 17.70 Median 69.14	Ease to use 12.24 Convenience 4.34 Discreetness 11.27 Psychological 9.84 Total 9.42	NR
Oh et al.	SF-36	NR	NR	Yes	PF 20.9 ± 2.4 RP 26.7 ± 3.2 RE 31.4 ± 3.6 VT 42.9 ± 1.8 MH 53.6 ± 1.8 SF 51.4 ± 2.3 BP 62.0 ± 2.4 GH 45.6 ± 1.5	NA	NA	NR
Pinder et al.	ISC-Q	Valid and reliable, understanding of the benefits of intermittent catheter devices	NR	Yes, validated in this study	*n* = 77 Ease to use 75.53 ± 17.87 Convenience 42.45 ± 23.83 Discreetness 54.82 ± 20.49 Psychological 62.77 ± 23.00 Total 61.65 ± 16.13	*n* = 77 Ease to use 71.63 ± 18.86 Convenience 41.48 ± 22.83 Discreetness 56.11 ± 20.67 Psychological 61.48 ± 23.81 Total 60.17 ± 15.50	NR	NR
Roberson et al.	ISC-Q	Questionnaire is validated	NR	Yes	ISC-Q Ease to use 82.0 Convenience 60.0 Discreetness 75.4 Psychological 64.3 Total 70.4	NA	NA	Incontinence (as measured by leakage in between catheterizations), number of UTIs, time performing ISC, race, gender, age, SCI causing ISC need, number of catheterizations per day, and type of catheter used (hydrophilic vs non-hydrophilic catheter)
Sekido et al.	SF-36 King’s Health Questionnaire	NR	NR	Yes	SF-36 (*n* = 50) PF 85.70 ± 17.10 RP 79.63 ± 26.24 BP 74.45 ± 22.46 GH 55.84 ± 15.20 VT 61.63 ± 18.21 SF 83.00 ± 19.84 RE 81.67 ± 25.37 MH 72.15 ± 18.86 PCS 45.26 ± 13.70 MCS 50.06 ± 8.97 Kings Health (*n* = 44) GHP 36.93 ± 19.05 II 37.88 ± 23.40 RL 22.76 ± 21.65 PL 25.00 ± 26.29 SL 15.04 ± 20.36 PR 10.29 ± 14.80 EM 33.33 ± 26.39 S/E 34.62 ± 25.76 SM 19.32 ± 20.73	NA	NA	NR
Svihra et al.	ICIQ-UI SF	NR	NR	Yes	Overall: 14.83 ± 4.10 (range: 4–21)	Overall: 9.12 ± 5.63 (range: 0–21)	Significant	NR
Wilde et al.	ISC-Q Psychological Well-being scale	NR	NR	Yes	13.02 ± 6.91	13.86 ± 6.45	Minus 0.19 ± 5.79 (*p* = 0.882)	NR
Yasami et al.	SF-36	Widely uses in SCI patients, reliable tool, validated in Persian for Iranian patients, frequently used in Iran	NR	Yes	IC-P PF 27.47 (18.37) RP 51.10 (29.07) RE 60.61 (34.69) MH 74.70 (17.28) VT 68.11 (13.50) SF 75.46 (21.22) BP 70.51 (22.68) GH 55.52 (27.93) PCS 57.52 (16.43) MCS 64.04 (16.61) Total score 61.00 (8.55) IC-A PF 20.25 (14.82) RP 46.20 (23.50) RE 41.66 (27.40) MH 68.22 (21.15) VT 60.21 (14.80) SF 64.16 (25.81) BP 59.82 (29.12) GH 48.39 (20.25) PCS 50.67 (21.17) MCS 56.53 (17.25) Total score 52.12 (7.99)	NA	NA	Higher SCI level, tetraplegia, shorter time since injury, and spontaneous micturition
Yeşil et al.	ISC-Q	Reliable and valid, easily self-administered, simplicity	NR	Yes	ISC-Q Ease of use Men: 62.5 ± 9.0 Women: 62.8 ± 8.6 ISC-Q Convenience Men: 62.7 ± 19.4 Women: 64.4 ± 17.3 ISC-Q Discreetness Men: 59.3 ± 12.1 Women: 52.5 ± 11.8 ISC-Q Psychological well-being Men: 68.1 ± 15.1 Women: 70.2 ± 14.6 ISC-Q Total Men: 63.4 ± 9.1 Women: 62.4 ± 9.2	NA	NA	NR
Yilmaz et al.	Five-point Likert scale	NR	NR	No	Effect on QoL: Much better Male 29 (14.6%) Female 5 (7.2%) Somewhat better Male 91 (46%) Female 37 (53.6%) About the same Male 52 (26.3%) Female 16 (23.2%) Somewhat worse Male 19 (9.6%) Female 10 (14.5%) Much worse Male 7 (3.5%) Female 1 (1.4%)	NA	NA	NR
Yoshida et al.	ISC-Q SF-12 Qualiveen	ISC-Q is well-validated for assessing the broader aspects of self-catheterization-related QoL, can be useful for understanding the benefits of intermittent catheter devices	NR	Yes	Reusable ISC-Q Ease of use: 63.0 ± 19.7 ISC-Q Convenience: 59.2 ± 30.5 ISC-Q Discreetness: 55.2 ± 20.9 ISC-Q Psychological well-being: 43.8 ± 27.7 ISC-Q Total: 53.5 ± 19.5 Single-use ISC-Q Ease of use: 59.9 ± 17.0 ISC-Q Convenience: 40.3 ± 21.1 ISC-Q Discreetness: 56.4 ± 25.9 ISC-Q Psychological well-being: 31.4 ± 18.9 ISC-Q Total: 46.9 ± 12.8 Subgroep follow-up *n* 30 ISC-Q Ease of use: 65.4 ± 18.3 ISC-Q Convenience: 57.8 ± 31.4 ISC-Q Discreetness: 54.1 ± 23.1 ISC-Q Psychological well-being: 42.4 ± 27.8 ISC-Q Total: 54.9 ± 19.4	Subgroup follow-up (*n* = 30) ISC-Q Ease of use: 69.6 ± 19.6 ISC-Q Convenience: 50.3 ± 28.1 ISC-Q Discreetness: 54.2 ± 20.6 ISC-Q Psychological well-being: 42.9 ± 27.0 ISC-Q Total: 54.2 ± 18.8	NR	NR
Zanollo et al.	SF-Qualiveen	NR	NR	Yes	Control group Inconvenience: 1.71 (1.21) Restrictions: 1.96 (0.73) Fears: 1.72 (1.18) Impact on daily life: 1.65 (1.23) Index: 1.76 (0.74) Study group Inconvenience: 1.58 (1.10) Restrictions: 1.91 (0.75) Fears: 1.56 (1.08) Impact on daily life: 1.30 (1.08) Index: 1.58 (0.59)	NR	NR	NR

BP, bodily pain; CIC, clean intermittent catheterization; CISC, Clean Intermittent Self-Catheterization; EM, emotions; EQ-5D, EuroQol 5D; EQ-5D VAS, EuroQol 5D Visual Analog Scale; GH, general health; GHP, General Health Perceptions; GHQ-28, General Health Questionnaire-28; IC-A, intermittent catheterization by an attendant/caregiver; IC-P, intermittent catheterization by patient; ICIQ-FLUTS, International Consultation on Incontinence Questionnaire Female Lower Urinary Tract Symptoms Modules; ICIQ-LUTS, International Consultation on Incontinence Modular Questionnaire-Lower Urinary Incontinence Quality of Life Module; ICIQ-SF, International Consultation on Incontinence Questionnaire-Short Form; ICIQ-UI SF, International Consultation on Incontinence Questionnaire-Urinary Incontinence Short Form; II, incontinence impact; I-QoL, Incontinence Quality of Life; ISC-Q, intermittent self-catheterization questionnaire; MCS, Mental Component Summary; MH, mental health; mSF36, This is the RAND36– Item Health Survey, with or without modification of the Physical Functioning Scale for those with spinal cord injury; NBSS, The Neurogenic Bladder Symptom Score; PCS, Physical Component Summary; PF, physical functioning; PL, physical limitations; PR, personal relationships; QBB, bowel/bladder; QFI, family/independence; QoL, quality of life; QUALAS-T, QUAlity of Life Assessment in Spina bifida for Teenagers; RE, role emotional; RL, role limitations; RP, role physical; SCI, spinal cord injury; SCI-QoL, Spinal Cord Injury Quality of Life; S/E, sleep/energy; SF, social functioning; SF-12, 12-Item Short-Form Health Survey; SF-36, 36-Item Short-Form Health Survey questionnaire; SF-Qualiveen, Short form of Qualiveen 30; SL, social limitations; SM, Severity measures; SS, symptom severity; SWLS, satisfaction with life scale; UTI, urinary tract infection; VAS, visual analog scale; VT, vitality; WHOQoL, World Health Organization Quality of Life.

#### Characteristics of study population

Eighteen studies solely included patients with SCI as an underlying cause for CIC, three studies only included patients with MS and one study solely included patients with spina bifida. The remaining studies comprised participants with various underlying diseases or only described if participants had a neurogenic or non-neurogenic cause to perform CIC. Nineteen studies included patients with a non-neurological disease, like (postoperative) retention, bladder outlet obstruction, or an underactive bladder The experience on CIC was reported in 29 studies and varied from starting CIC at the time of inclusion to a median of 10.5 years. One study included female participants exclusively and one study included only male patients. All other studies included a mixed population. The mean age of the participants varied from 31.8 to 62.0 years, five studies did not report the age of the participants other than they were adults at the time of inclusion and one study only reported that the age of participants ranged from 14 to 20 years old. Only seven studies reported on the mean age when CIC was started, which varied from 35.2 (±20.5) to 62 (±13.4) years. Patients with diversions were excluded in five studies and were analyzed separately in five other studies. One study included patients with a diversion but did not analyze the QoL separately for this group. The majority of the studies (32) did not report whether or not patients with a diversion or non-urethral catheterization (continent catheterizable tubes/stomas) were included in their population.

#### Reported information on catheters and CIC

Thirteen studies reported which catheters were used in their included population, which can be found in Table 2. One of these studies described the used catheter as discreet, but no further explanation on which type of catheter was given. Twelve studies provided information about their participants using single-use or reusable catheters. In seven studies only single-use catheters were used, in the other five studies reusable catheters were also used. The frequency of catheterization per 24 h was reported in 12 studies and varied from ⩽1 times per 24 h to >6 times per 24 h. Six studies described a mean frequency, which varied between 3 and 6 times per 24 h.

Five studies explicitly described that CIC was performed by the included patients themselves, one study described that it was performed by a third party in all patients and 15 studies reported that both CIC by the patients themselves or a third party occurred. The remaining 25 studies did not provide information on this. UTIs in the study population were reported in various ways in 14 studies and the presence of comorbidities was reported in 7 studies (Table 2).

### Results on outcome parameters

#### Used tools to measure QoL

A total of 21 different questionnaires/tools were used to measure QoL in patients on CIC in the included studies (Table 3). The characteristics of these tools and how often they were used in the included studies can be found in Table 4. The 36-Item Short-Form Health Survey questionnaire (SF-36) was the most frequently used tool, namely in nine of the included studies. The Intermittent self-catheterization questionnaire (ISC-Q) was used to measure QoL in eight of the included trials. Twelve included studies used two or more tools to measure QoL. Three studies used tools that are not validated, James et al. used a 7-point Likert scale questionnaire that was developed for the database specifically,^27^ Kessler et al. used a questionnaire that was based on the validated 12-item Short-Form Health Survey (SF-12)^28^ and Yilmaz et al. used a five-point Likert scale on the effect of CIC on QoL.^47^

**Table 4.** Used tools to measure QoL, abbreviations of the questionnaires, what is measured, their normal ranges, and how often they were used in the included studies in this systematic review.

Used tool	Abbreviation	What is measured	Normal range	Times used
36-Item Short-Form Health Survey questionnaire	SF-36	Eight scales: PF, RP, BP, GH, VT, SF, RE, and MH. Two summary measures can be calculated from these scales—these are called the physical component score and the mental component score.	To score the SF-36, scales are standardized with a scoring algorithm or by the SF-36v2 scoring software to obtain a score ranging from 0 to 100. Higher scores indicate better health status and a mean score of 50 has been articulated as a normative value for all scales.	9
Intermittent self-catheterization questionnaire	ISC-Q	Aspects of QoL in four domains: Ease of use Convenience Discreetness Psychological well-being.	Domain scores : 0–100 scale, with a higher score representing less burden associated with ISC.	8
King’s Health Questionnaire	KHQ	The KHQ contains two single-item questions to address GHP and II, and the following seven multi-item domains: RL, PL, SL, PR, EM, S/E, and (I)SM.	Range of domain scores: 0 (best) to 100 (worst). Range of the symptom severity scale score: 0 (best) to 30 (worst). Lower score = better QoL.	5
12-item Short-Form Health Survey (an abbreviated version of the SF-36)	SF-12	Eight scales: PF, RP, BP, GH, VT, SF, RE, and MH. Two summary measures can be calculated from these scales—these are called the physical component score and the mental component score.	To score the SF-12, scales are standardized with a scoring algorithm. Scores range from 0 to 100, with higher scores indicating better physical and MH functioning.	5
Visual Analog Scale (on QoL)	VAS	VAS can be presented in numerous ways, numerical rating scales, curvilinear analog scales, box scales, graphic rating scales, etc.	Dependent on presented scale.	4
Qualiveen 30	NA	30 Items focusing on 4 aspects of patient lives, including bother with limitations (9 items), frequency of limitations (8), fears (8), and feelings (5). Response options are framed as 5-point Likert-type scales with 0 indicating no impact of urinary problems on HRQoL and 4 indicating a high adverse impact of urinary difficulty on HRQoL.	Qualiveen domain scores are calculated as an average of the scores on items in that domain and, thus, the range is 0–4 with an overall score representing the mean of the four domains, which also ranges from 0 to 4.	4
Short form of Qualiveen 30	SF-Qualiveen	SF-Qualiveen, which is self-administered, is composed of eight items distributed in four domains, including bother with limitations (two items), frequency of limitations (2), fears (2), and feelings (2). Responses are given on a 5-point Likert-like scale, where a score of 0 indicates “no impact” and 4 “high impact.”	The SF-Qualiveen total score is calculated as the mean of the eight responses and the domain scores are calculated as the mean score of the responses per domain.	3
General Health Questionnaire-28	GHQ-28	The questionnaire consists of four sub-components that measure public health (somatic symptoms, anxiety and insomnia, social dysfunction, and depression), and each component consists of seven questions.	GHQ scoring methods are based on the Likert scale from zero to three and a lower score indicates a better mental state. Minimum, maximum, and cut-off scores are 0, 84, and 24, respectively.	3
World Health Organization Quality of Life, abbreviated version of the WHOQoL-100 quality of life assessment	WHOQoL-Bref	Four domains related to QoL: physical health, psychological, social relationships, and environment. It also includes one facet on the overall QoL and GH.	Domain scores for the WHOQoL-Bref are calculated by multiplying the mean of all items included within the domain by four. Potential scores for all domain scores, therefore, range from 4 to 20.	3
The Neurogenic Bladder Symptom Score	NBSS	24-Item questionnaire that measures bladder symptoms across three different domains: incontinence, storage and voiding, and consequences; there is a single general urinary QoL question.	Incontinence (scored 0–29). Storage and voiding (scored 0–22). Consequences (scored 0–23). General urinary QoL question scored from 0 (pleased) to 4 (unhappy). For all domains, a higher score represents a worse symptom burden or QoL.	2
International Consultation on Incontinence Questionnaire-Urinary Incontinence Short Form	ICIQ-SF/ICIQ-UI SF	A questionnaire for evaluating the frequency, severity, and impact on QoL of urinary incontinence in men and women. It consists of four scoring items: frequency or urinary incontinence, amount of leakage, the overall impact of urinary incontinence, and a self-diagnostic item.	First question is scored 0–5, second one is scored either 0, 2, 4, or 6, and the final one is scored on a Likert scale from 0 to 10. Overall scores range from 0 to 21, higher scores indicate greater impact of symptoms.	2
Perceived health status Satisfaction with life scale	SWLS	SWLS consists of five items all tapping into global life satisfaction: (1) in most ways my life is close to my ideal; (2) the conditions of my life are excellent; (3) I am satisfied with my life; (4) so far I have gotten the important things I want in life; and (5) if I could live my life over, I would change almost nothing. 7-Point Likert scale is used ranging from 1 “Strongly disagree” to 7 “Strongly agree.”	Scores consist of a raw score (between 5 and 35). Higher scores represent higher life satisfaction. Scorers can be assigned into six well-being categories and interpretative text in provided for each. 30–35 Extremely satisfied 25–29 Satisfied 20–24 Slightly satisfied 15–19 Slightly dissatisfied 10–14 Dissatisfied 5–9 Extremely dissatisfied.	1
Incontinence Quality of Life	I-QoL	Three subscales: Avoidance and limiting behaviors, psychosocial impacts, and social embarrassment.	The 22 items in the I-QoL are summed and then transformed to a 0–100 scale for greater interpretability, with the higher scores representing greater QoL. The I-QoL subscales are scored in an identical manner.	1
QUAlity of Life Assessment in Spina bifida for Teenagers	QUALAS-T	Two domains: family and independence, bladder and bowel, with five questions per domain. Each domain consists of five items (10 items in total), and each item was measured on a five-point Likert scale: (1) never (no points), (2) almost never (5 points), (3) sometimes (10 points), (4) almost always (15 points), and (5) always (20 points).	The total score of each domain ranges from 0 to 100 points, and the higher the score, the higher the HRQoL.	1
Semiannual NARCOMS questionnaire 7-Point Likert scale (indicating change due to treatment)	NA	Each semiannual NARCOMS questionnaire includes an assessment of disability status and QoL, participants are asked if their QoL had changed with treatment (7-point Likert scale, 1 much better, 7 much worse).	Likert score = 1–3: Very negatively/negatively/slightly negatively. Likert score = 4: Neutral. Likert score = 5–7: Very positively/positively/slightly positively.	1
International Consultation on Incontinence Modular Questionnaire-Lower Urinary Incontinence Quality of Life Module	ICIQ-LUTS	The ICIQ-LUTSqol is a psychometrically robust patient-completed questionnaire evaluating QoL in urinary incontinent patients. The ICIQ-LUTSqol is the KHQ adapted for use within the ICIQ structure and provides a measure to assess the impact of urinary incontinence on QoL with particular reference to social effects. This tool explores the impact on patients’ lives of urinary incontinence and can be used as an outcome measure to assess the impact of different treatment modalities.	All questions are scored 1–4. Overall scores are between 19 and 76, with greater values indicating increased impact on QoL. Other scales are not incorporated in the overall score but indicate the impact of individual symptoms on the patient.	1
International Consultation on Incontinence Questionnaire Female Lower Urinary Tract Symptoms Modules	ICIQ-FLUTS	The ICIQ-FLUTS is composed of three domains that represent storage (or filling, F score), voiding (V score), and incontinence (I score) symptoms. The instrument has 12 items with two questions for each item. The first question asks about the presence and severity of a symptom, and respondents quantify the symptom using a Likert scale (0 = never; 4 = all the time). The second question determines the degree to which the symptom affects the individual, with scores from 0 (not at all) to 10 (a great deal).	Each question is scored 1–4; thus, range of overall scores from 0 to 16, 12, and 20 for filling, voiding and incontinence scales, respectively. Overall scores are between 0 and 48 where all subscale scores are added. Higher scores indicate a greater impact on individual symptoms for the patient.	1
EuroQol 5D	EQ-5D	The EQ-5D-3L/5L descriptive system comprises the following five dimensions: mobility, self-care, usual activities, pain/discomfort, and anxiety/depression. Each dimension has three or five levels. The patient is asked to indicate his/her health state by ticking the box next to the most appropriate statement in each of the five dimensions.	The given answers result in a 1-digit number that expresses the level selected for that dimension. The digits for the five dimensions can be combined into a 5-digit number that describes the patient’s health state.	1
EuroQol 5D Visual Analog Scale	EQ-5D VAS	The EQ VAS records the patient’s self-rated health on a vertical VAS where the endpoints are labeled “Best imaginable health state” and “Worst imaginable health state.” The VAS can be used as a quantitative measure of health outcome that reflects the patient’s own judgment.	Outcome ranges from 0 (worst imaginable health state) to 100 (best imaginable health state).	1
Spinal Cord Injury Quality of Life Measurement System Bladder Management Difficulties	SCI-QoL difficulties score	The SCI-QoL consists of many different item banks, which were validated in individuals with SCI, to assess multiple aspects of the health and psychosocial impact of SCI. The SCI-QoL Difficulties item bank, assesses the ability to perform a bladder program, incontinence concerns, and impacts on daily life.	The SCI-QoL questionnaires rely on item response theory and computer adaptive testing, which enables the questionnaire to adapt to participant answers. The SCI-QoL item banks have a mean score of 50 and a range of 0–100 with lower scores indicating less bladder difficulty.	1
Five-point Likert scale on QoL	NA	To assess how IC affects the QoL, a five-point Likert scale was used: 1: Much better, 2: Somewhat better, 3: About the same, 4: Somewhat worse, 5: Much worse.	NA	1

BP, bodily pain; EM, emotions; EQ-5D, EuroQol 5D; GH, general health; GHP, general health perceptions; GHQ-28, General Health Questionnaire-28; IC, intermittent catheterization; HRQoL, health-related quality of life; II, incontinence impact; (I)SM, (incontinence) severity measures; MH, mental health; PF, physical functioning; PL, physical limitations; PR, personal relationships; QoL, quality of life; RE, role emotional; RL, role limitations; RP, role physical; SCI, spinal cord injury; S/E, sleep/energy; SF, social functioning; SL, social limitations; VT, vitality.

One patient reported outcome measure (PROM) was developed to measure QoL in neurogenic patients on CIC (ISC-Q), others are suitable to measure general QoL (SF-36, King’s Health Questionnaire (KHQ), SF-12, Visual Analog Scale (VAS), General Health Questionnaire-28 (GHQ-28) World Health Organization Quality of Life (WHOQoL)-Bref, satisfaction with life scale (SWLS), EuroQol 5D (EQ-5D), EuroQol 5D Visual Analog Scale (EQ-5D VAS), and five-point Likert scale on QoL), developed for neurogenic bladder dysfunction (Qualiveen 30, SF-Qualiveen, The Neurogenic Bladder Symptom Score (NBSS), QUAlity of Life Assessment in Spina bifida for Teenagers (QUALAS-T), and Spinal Cord Injury Quality of Life (SCI-QoL) difficulties score) or were mainly incontinence oriented (International Consultation on Incontinence Questionnaire-Urinary Incontinence Short Form (ICIQ-SF/ICIQ-UI SF), Incontinence Quality of Life (I-QoL), International Consultation on Incontinence Modular Questionnaire-Lower Urinary Incontinence Quality of Life Module (ICIQ-LUTS), and International Consultation on Incontinence Questionnaire Female Lower Urinary Tract Symptoms Modules (ICIQ-FLUTS)).

#### Measured overall QoL and predictors of QoL

As shown in Tables 3 and 4 the way of reporting on QoL between the used tools differs tremendously. Therefore, no statement/qualification on the overall QoL in patients on CIC can be made or given.

Nine studies reported on possible predictors that contribute to the measured QoL in patients on CIC (Table 3). Some predictors that were mentioned are: UTIs, level of SCI, the occurrence of spontaneous micturition or incontinence, frequency of CIC, type of catheter, third-party CIC/independence, and pain during catheterization.

#### Measured QoL dependent on type of catheter

There was a lack of information on the relationship between the used type of catheter and QoL in most of the studies, so no conclusions on this could be drawn.

### Subgroup analysis

Due to several reasons, it was not possible or contributive to perform a subgroup analysis for all predefined subgroups. First, a large heterogeneity of the studied population was seen in the included studies. This was the case for age, gender, cause for catheterization, and whether or not patients with non-urethral catheterization were included. Second, there was also a great variety seen in the used tools/questionnaires to measure QoL. The outcomes of these tools/questionnaires differed widely, which made comparison of QoL between the different measures, impossible.

Instead of performing an analysis of subgroups, the usage of different QoL measurements within these subgroups will be described.

#### Men and women

The majority of the studies included mixed-sex populations, one study only included female patients, and one study only included male patients. Therefore, no conclusions can be drawn about the used questionnaires in sex-specific populations.

#### Pediatric/adolescent patients

Only one study included patients of mostly adolescent age (14–20 years of age).^25^ The authors used the QUALAS-T questionnaire,^50^ which is validated in a population of 13–17 years old.

#### Neurogenic and non-neurogenic causes for catheterization

The studies that included a mixed population of patients that are on CIC due to neurogenic and non-neurogenic causes, did not report the QoL of these groups separately. Eighteen studies solely included patients with SCI; no uniformity was seen in the used questionnaires in this population, as 10 different tools to measure QoL were used (Table 3).

#### Diversion or non-urethral catheterization (continent catheterizable tubes/stoma’s)

The five studies^14,16,22,23,32^ that included patients with a diversion or with non-urethral catheterization all used different tools/measurements to report on QoL: SF-36, ICIQ-SF, NBSS, SF-12, and I-QoL. No validation of these measurements in this specific patient population could be found in the current literature.

### Risk of bias analysis

**Figure 2.**
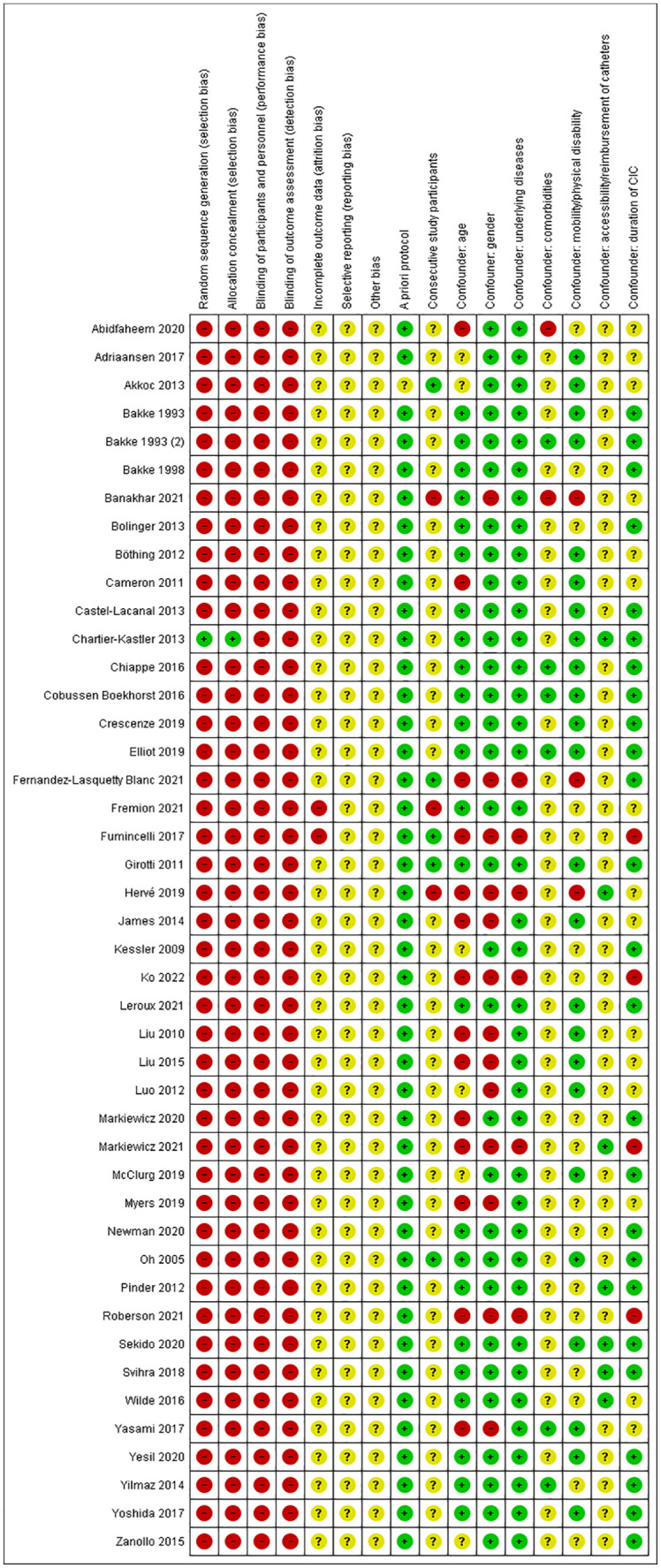
Risk of bias summary.

The Cochrane Risk of Bias Assessment tool was used to assess the risk of bias in the included studies. Bias and the predefined possible confounding factors in these studies were classified as high (−), unclear (?), or low (+). Bias was assessed as high or unclear in most studies. The risk of confounding factors was scored as low or unclear in most studies. A summary of the findings of this assessment can be found in Figure 2.

## Discussion

CIC is the golden standard treatment for patients with neurogenic and non-neurogenic LUTD leading to bladder emptying difficulties.^3,4^ In the Netherlands alone there were 46,000 CIC users in 2018^51^ and worldwide there are millions of people that self-catheterize.^1^ Evaluation of their QoL and identifying possible challenges that these patients face can help to improve the standard of care and their QoL. Using patient reported outcome measures (PROMS) can also be helpful in initiating conversations between patients and caregivers about certain aspects, like QoL.

This systematic review showed a great variety in the used tools/measurements and reported QoL of patients on CIC. In the 43 included studies, 21 different tools were used. There are a lot of tools available to measure QoL, one was specifically developed and validated in patients who perform CIC (ISC-Q), others are suitable to measure general QoL (SF-36, KHQ, SF-12, VAS, GHQ-28 WHOQoL-Bref, SWLS, EQ-5D, EQ-5D VAS, and five-point Likert scale on QoL), some other tools were developed for neurogenic bladder dysfunction (Qualiveen 30, SF-Qualiveen, NBSS, QUALAS-T, and SCI-QoL difficulties score) or were more incontinence oriented (ICIQ-SF/ICIQ-UI SF, I-QoL, ICIQ-LUTS, and ICIQ-FLUTS). The SF-36 was the most frequently used questionnaire. This questionnaire was designed for use in clinical practice and research, health policy evaluations, and general population surveys. It consists of one multi-item scale that assesses eight health concepts and is a generic instrument to measure Health Related Quality of Life (HRQoL).^52^ The SF-12, which was used five times in this review, is an abbreviated version of the SF-36 and measures the same concept.^53^ The second most used questionnaire was the ISC-Q, which was developed to specifically evaluate QoL in patients who are on CIC. Validation was done in patients with urinary retention due to a neurogenic cause.^40^ A systematic review of QoL performed by Pequeno et al. in 2020 in adult patients on CIC-assessed instruments in population-based studies around the world. The authors debate that heterogeneity of the used tools hinders comparison between similar populations and that further research is necessary to determine which QoL instrument is best suited in which situation.^51^

As seen in earlier research performed by our group, the majority of the patients on CIC have a non-neurogenic underlying cause.^54^ Most studies in this systematic review included a mixed neurogenic and non-neurogenic population or solely a neurogenic population. The measured QoL was not reported separately in the mixed populations. The distinction is important because a neurogenic population differs from a non-neurogenic population, with more comorbidities and other factors contributing to QoL like a possible dependency on caregivers. Thus, separate reporting of QoL of neurogenic and non-neurogenic patients is advisable.

It was challenging to identify the scoring methods and normal ranges of each questionnaire/measurement tool. These normal ranges and measurement properties differed, as shown in Table 4. It was easily confirmed in the literature if the used tools were validated, but it could not always be confirmed that the tools were validated for use in this specific patient population or to confirm that the tool was administered the correct way.

In the current literature, there are multiple comparable systematic reviews. In 2017 Fumincelli et al.^5^ conducted a scoping review on the QoL of patients on intermittent catheterization and their caregivers. They included 13 studies with a neurogenic bladder patient population and compared the main results and conclusions of these studies, rather than looking at the measured outcome of the used tools. They found that patients on CIC had lower QoL compared to patients with normal bladder function. When good pain control during the procedure was encouraged, better QoL scores were observed, particularly in the social and psychological domains. Dependency on caregivers for performing CIC was the main factor negatively affecting the patient’s QoL. Wang et al.^55^ performed a systematic review evaluating PROMS in CIC users. They found the ISC-Q as the most widely used questionnaire to measure HRQoL in patients on CIC but emphasized the need to perform translation and validation studies and cross-cultural adaptations to allow usage of the tool by professionals and patients in their own language and in different countries/societies/cultures. Clark and Welk^56^ performed a systematic review regarding the used PROMS in neurogenic patients and found only a few tools that were specifically developed for this population. They underlined the need for understanding the principles of PROM development and systematic evaluation, so clinicians and researchers can select the most suitable PROM for their specific purpose. In our systematic review, we included all patients on CIC in whom QoL was measured, regardless of their underlying cause, and checked if the used tools were used in the correct population, which resulted in a complete and critical overview of all tools used.

This systematic review was conducted according to a premade and preregistered protocol that conforms to the Cochrane Handbook for Systematic Reviews of interventions^57^ and PRISMA guidelines.^6^ It gives an overview of the current literature available on the used measurements to evaluate QoL in patients on CIC. There are however some limitations. First, the predefined subgroup analysis could not be performed due to the heterogeneity of the patient populations, the used tools, and their outcome measures. Second, our search was limited to only English-written publications. Lastly, our research question was quite broad because we aimed to include both neurogenic and non-neurogenic patients, resulting in a high yield of articles. A more specified search strategy regarding a specific patient population might have led to a feasible overall QoL analysis in that population.

## Conclusion

The 43 included studies showed the variety of used tools to measure QoL in patients on CIC. Because of the lacking uniformity of the measured QoL outcomes, various included studies could not be compared and subgroup analysis was impossible. Only one measurement tool was developed for evaluating QoL in neurogenic bladder patients on CIC. In a future perspective, it is advised to develop or validate an existing PROM that can measure QoL in patients on CIC explicitly and is validated in both a neurogenic and a non-neurogenic patient population and to perform translation and validation studies for cross-cultural adaptation. Furthermore, it is advised to report QoL of neurogenic and non-neurogenic patients separately.

Lastly, the scoring system must be transparent and easily used to enable smooth implementation in clinical practice and research as complicated tools are more challenging to carry out in daily practice. This enables clinicians and researchers to compare different treatment options and studies and leads to a better insight into QoL in patients on CIC and their challenges.

## Supplemental Material

sj-docx-1-tau-10.1177_17562872241303447 – Supplemental material for Quality of life aspects of intermittent catheterization in neurogenic and non-neurogenic patients: a systematic review on heterogeneity in the measurements used Supplemental material, sj-docx-1-tau-10.1177_17562872241303447 for Quality of life aspects of intermittent catheterization in neurogenic and non-neurogenic patients: a systematic review on heterogeneity in the measurements used by Tess van Doorn, Rosa L. Coolen, Jan Groen, Jeroen R. Scheepe and Bertil F. M. Blok in Therapeutic Advances in Urology

sj-docx-2-tau-10.1177_17562872241303447 – Supplemental material for Quality of life aspects of intermittent catheterization in neurogenic and non-neurogenic patients: a systematic review on heterogeneity in the measurements used Supplemental material, sj-docx-2-tau-10.1177_17562872241303447 for Quality of life aspects of intermittent catheterization in neurogenic and non-neurogenic patients: a systematic review on heterogeneity in the measurements used by Tess van Doorn, Rosa L. Coolen, Jan Groen, Jeroen R. Scheepe and Bertil F. M. Blok in Therapeutic Advances in Urology

## Declarations

**Ethics approval and consent to participate:** Not applicable.

**Consent for publication:** Not applicable.

**Availability of data and materials:** The dataset used is available from the corresponding author on reasonable request.
