# Supplementary material for: Quality of life aspects of intermittent catheterization in neurogenic and non-neurogenic patients: a systematic review on heterogeneity in the measurements used
Source: Ther Adv Urol. 2024 Dec 23;16:17562872241303447. doi: 10.1177/17562872241303447 (PMC11664527; doi:10.1177/17562872241303447)
Supplement: sj-docx-1-tau-10.1177_17562872241303447 – Supplemental material for Quality of life aspects of intermittent catheterization in neurogenic and non-neurogenic patients: a systematic review on heterogeneity in the measurements used [file sj-docx-1-tau-10.1177_17562872241303447.docx]

**Attachment 1:** used search string.

| **Database searched** | **via** | **Years of coverage** | **References** | **After de-duplication** |
| --- | --- | --- | --- | --- |
| Embase | Embase.com | 1971 - Present | 3187 | 3159 |
| Medline ALL | Ovid | 1946 - Present | 1648 | 511 |
| Web of Science Core Collection * | Web of Knowledge | 1975 - Present | 936 | 156 |
| Cochrane Central Register of Controlled Trials | Wiley | 1992 - Present | 235 | 64 |
| CINAHL | EBSCO | 1982 - Present | 250 | 53 |
| Other sources: Google Scholar | | | 200 | 93 |
| **Total** | | | **6456** | **4036** |

*Science Citation Index Expanded (1975-present) ; Social Sciences Citation Index (1975-present) ; Arts & Humanities Citation Index (1975-present) ; Conference Proceedings Citation Index- Science (1990-present) ; Conference Proceedings Citation Index- Social Science & Humanities (1990-present) ; Emerging Sources Citation Index (2015-present)

**Embase.com**

('quality of life'/de OR 'quality of life assessment'/exp OR 'satisfaction'/de OR 'patient satisfaction'/de OR ((quality NEAR/3 life) OR hrqol OR HRQL OR satisf* OR (patient* NEAR/3 (perception* OR perceive* OR experience*))):ab,ti,kw) AND ('bladder catheterization'/exp OR 'intermittent catheterization'/de OR 'ureter catheterization'/de OR ('catheterization'/de AND ('neurogenic bladder'/de OR 'self care'/de)) OR (((intermit* OR self* OR Mitrofanoff* OR non-urethral* OR continent*) NEAR/3 (catheter*))):ab,ti,kw)

**Medline (Ovid)**

(Quality of Life/ OR Personal Satisfaction/ OR Patient Satisfaction/ OR ((quality ADJ3 life) OR hrqol OR HRQL OR satisf* OR (patient* ADJ3 (perception* OR perceive* OR experience*))).ab,ti,kf.) AND (exp Urinary Catheterization/ OR (Catheterization/ AND (Urinary Bladder, Neurogenic/ OR Self Care/)) OR (((intermit* OR self* OR Mitrofanoff* OR non-urethral* OR continent*) ADJ3 (catheter*))).ab,ti,kf.)

**Web of Science**

TS=((((quality NEAR/2 life) OR hrqol OR HRQL OR satisf* OR (patient* NEAR/2 (perception* OR perceive* OR experience*)))) AND ((((intermit* OR self* OR Mitrofanoff* OR non-urethral* OR continent*) NEAR/2 (catheter*)))))

**Cochrane Central**

(((quality NEAR/3 life) OR hrqol OR HRQL OR satisf* OR (patient* NEAR/3 (perception* OR perceive* OR experience*))):ab,ti,kw) AND ((((intermit* OR self* OR Mitrofanoff* OR non-urethral* OR continent*) NEAR/3 (catheter*))):ab,ti,kw)

**Cinahl**

(MH "Quality of Life" OR MH "Personal Satisfaction" OR MH "Patient Satisfaction" OR AB ((quality N2 life) OR hrqol OR HRQL OR satisf* OR (patient* N2 (perception* OR perceive* OR experience*))) OR TI ((quality N2 life) OR hrqol OR HRQL OR satisf* OR (patient* N2 (perception* OR perceive* OR experience*)))) AND (MH "Urinary Catheterization"+ OR (MH Catheterization AND (MH "Bladder, Neurogenic" OR MH "Self Care")) OR AB (((intermit* OR self* OR Mitrofanoff* OR non-urethral* OR continent*) N2 (catheter*))) OR TI (((intermit* OR self* OR Mitrofanoff* OR non-urethral* OR continent*) N2 (catheter*))))

**Google Scholar (top 200 ranked on relevance)**

“quality life”|hrqol|HRQL|satisfaction “intermittent|Mitrofanoff|continent catheter|catheterization|catheterisation”
